# Congestion Pricing for Aircraft Pushback Slot Allocation

**DOI:** 10.1371/journal.pone.0170553

**Published:** 2017-01-23

**Authors:** Lihua Liu, Yaping Zhang, Lan Liu, Zhiwei Xing

**Affiliations:** 1 School of Transportation Science and Engineering, Harbin Institute of Technology, Harbin, Heilongjiang, China; 2 School of Civil and Transportation Engineering, Henan University of Urban Construction, Pingdingshan, Henan, China; 3 School of Safety and Environment Engineering, Hunan Institute of Technology, Hengyang, China; 4 Ground Support Equipment Research Base, Civil Aviation University of China, Tianjin, China; Beihang University, CHINA

## Abstract

In order to optimize aircraft pushback management during rush hour, aircraft pushback slot allocation based on congestion pricing is explored while considering monetary compensation based on the quality of the surface operations. First, the concept of the “external cost of surface congestion” is proposed, and a quantitative study on the external cost is performed. Then, an aircraft pushback slot allocation model for minimizing the total surface cost is established. An improved discrete differential evolution algorithm is also designed. Finally, a simulation is performed on Xinzheng International Airport using the proposed model. By comparing the pushback slot control strategy based on congestion pricing with other strategies, the advantages of the proposed model and algorithm are highlighted. In addition to reducing delays and optimizing the delay distribution, the model and algorithm are better suited for use for actual aircraft pushback management during rush hour. Further, it is also observed they do not result in significant increases in the surface cost. These results confirm the effectiveness and suitability of the proposed model and algorithm.

## Introduction

Congestion and delays are problems that occur routinely at domestic and international airports and not only reduce the operational efficiency but also result in huge losses to the airlines. The increase in air transportation demands at airports can no longer be satisfied simply by constructing more infrastructure, given the constraints of limited resources and funds. Hence, demand management has become an essential policy for the future.

“Congestion pricing” is the most direct pricing approach for resolving the issue of the mismatch between capacity and demand during airport operation. Under this strategy, grandfather rights are abandoned, and a congestion-based system with fees that vary depending on the degree of congestion is set up by the administrative department [1]. Odoni explored the problems encountered under actual conditions owing to institutional and other constraints [2]. Johnson and Savage calculated the congestion toll to be paid based on an analysis of the relationship between the number of departures and their respective delays. They found that the toll falls with a decrease in the length of the departure queue, and aircraft are only charged for the delays caused to the subsequently departing flights and not all flights [3]. Flores-Fillol was the first to explore the impact of congestion pricing on aircraft size and flight frequency and reported that tolls can result in carriers operating larger aircraft at lower frequencies [4]. Zhang et al. studied the decisions made by airport authorities regarding charges and capacity and proposed a model for maximizing profit with the aim of making the fees equal to the social marginal cost [5]. Avenali et al. developed an administered incentive pricing model based on the regulation of the radio spectrum and computed the marginal value for each slot by determining the failures in service [6]. Liu performed preliminary calculations on the price of pushback slots but did not take into account external costs [7]. However, even though “congestion pricing” at airports has been explored since the beginning of this century, it has not found wide acceptability. The effects of a market economy and economic measures on congestion pricing remain to be explored. Most studies on congestion pricing have focused on the pricing of airport resources, and there have been few studies on specific pricing mechanisms for individual aircraft. However, the above-mentioned studies on pricing strategy, cost analysis, and decision-making can serve as references for the congestion pricing of pushback resources.

Another approach is to use surface operation management techniques; these can include ground holding strategies [8], taxiing route optimization techniques [9], and aircraft pushback management methods. Atkin proposed a pushback time allocation method for the optimization of the departure sequence at rush hour, highlighting the potential benefits of pushback time control [10]. Wang et al. designed a pushback decision strategy with the objective of minimizing the total delay [11]. Jason described a two-sequence-dependent separation problem between takeoff and pushback sequencing [12]. Simaiakis proposed the concept of the “pushback rate” [13] and determined the suggested rate, applying it at Boston Logan International Airport [14]. Sandberg developed a decision support system and field tested it at Boston Logan Airport [15]. Fornés analyzed the gate-holding limits [16].

Currently, aircraft pushback in China remains based on grandfather rights, and the “first come, first service” strategy is employed. However, grandfather rights can frequently result in significant delays in access to resources. Further, the “first come, first serve” strategy is likely lead to congestion because of the concentration of aircraft at pushback time. A pushback control strategy can be adopted to disperse the aircraft at pushback time. Meanwhile, proper measures should be implemented to reduce delays caused by gate holding. Recent efforts to optimize aircraft scheduling have shown that the correct pricing mechanism can motivate airlines to transfer low-efficiency flights to off-peak hours [17]. This can lead to a new method for aircraft pushback that involves pushback control and a charge for using the resources.

On the basis of previous studies and by combining pushback control with a demand management strategy, we introduce the concept of pushback slots in this study and determine the price of each slot to compensate for congestion loss, in order to improve congestion and reduce delays as well as optimize the allocation of resources. First, based on existing research on traffic congestion, the concept of the “external cost of surface congestion” is introduced and a method for calculating it is proposed. Then, a cost-minimization aircraft pushback slot allocation model is established based on an analysis of the surface cost for all aircrafts and the constraints related to pushback slots allocation. Next, an improved discrete differential evolution algorithm (IDDE) is developed by improving the differential mutation and crossover operation of the conventional differential evolution algorithm. Finally, a simulation analysis is performed at Xinzheng International Airport, in order to confirm the feasibility and determine the effectiveness of the proposed model and algorithm.

## Model and Methods

### External cost of surface congestion

There is a considerable amount of existing research on traffic congestion pricing. Chen and Zhang proposed a congestion pricing model that aimed at maximizing the overall efficiency of traffic networks and developed a related genetic algorithm [18]. Zhang and Ma developed a self-adaptive tolling strategy for high-occupancy toll lane systems and performed a detailed simulation [19]. The above-mentioned studies on traffic congestion pricing can serve as references for analyzing the external cost of surface congestion.

With reference to the external cost of traffic congestion [20], the external cost of surface congestion can be defined as follows: the fee that an aircraft should but does not pay as monetary compensation to the airport for affecting the operational quality of other aircraft. This fee can be used for infrastructure construction, which would also aid the sustainable development of airport surface resources. By definition, the external cost is the basis for pushback slot pricing and should be equal to the total price.

**Fig 1:**
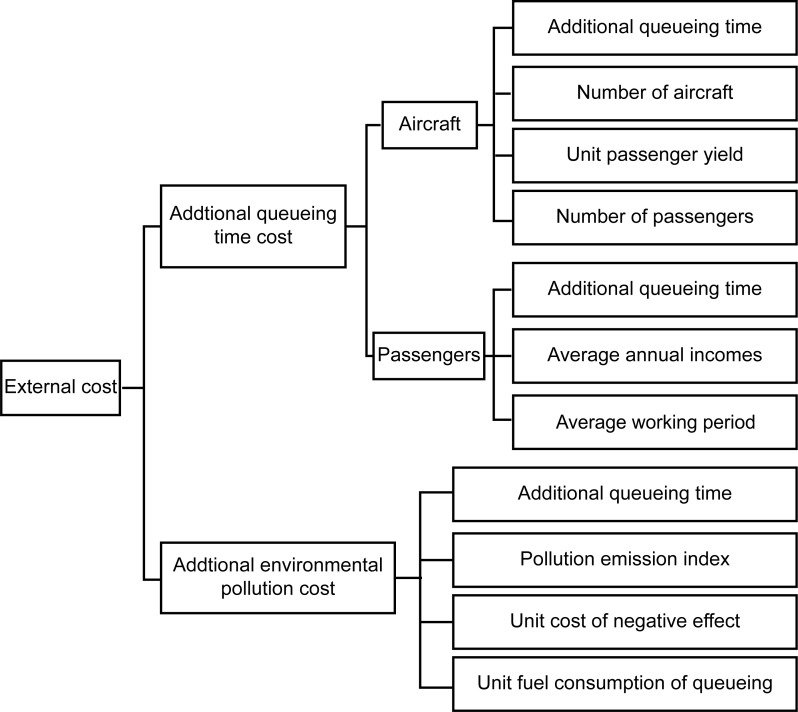
External cost resulting from congestion.

Ignoring the conflicts related to taxiing, the externality of congestion can be defined in terms of the increases in the queuing time and environmental pollution. Then, the external cost can be divided into the additional queuing time-related cost and the additional environmental pollution-related cost, as shown in Fig 1.

(1) Additional queuing time cost. Considering the additional time value of aircraft and passengers, the additional queuing time cost can be calculated as follows:

$$C_{queue}=T_{queue}\times \left(U_{ac}+U_{p}\right)=\sum_{i=1}^{n}\left(t_{i,queue}\right)\times \left[\frac{1}{n}\sum_{i=1}^{n}\left(p_{i,\;ac}/t_{i,\;ac}\right)+\left(I_{p}/H_{p}\right)\sum_{i=1}^{n}\left(x_{i}\times p_{i}\right)\right]$$ (1)

where *C*_*queue*_ is the additional queuing time cost (Chinese Yuan, CNY); *T*_*queue*_ is the additional queuing time (min); *U*_*p*_ is the unit passenger time value (CNY/min); *U*_*ac*_ is the unit aircraft time value (CNY/min); *i* is the type of aircraft, *i* = 1,2,⋯,*n*; *t*_*i*,*queue*_ is the additional queuing time (min); *x*_*i*_ is the aircraft number; *p*_*i*_ is the average number of passengers; *I*_*p*_ is the average annual income of the passengers (CNY); *H*_*p*_ is the average working period of the passengers (min); *p*_*i*,*ac*_ is the average airfare (CNY/person); and *t*_*i*,*ac*_ is the average running time (min).

(2) Additional environmental pollution cost. The primary pollutants in aircraft engine emissions are nitrogen oxide (NO_x_), carbon monoxide (CO), and unburned hydrocarbons. The additional environmental cost can be calculated using the following formula:

$$C_{Envi}=T_{queue}\times U_{poll}=\sum_{i=1}^{n}\left(t_{i,queue}\right)\times \left[c_{D}\times c^{taxi}\times \sum_{u=1}^{3}\left(c_{u,\;p}\right)\right]$$ (2)

where *C*_*Envi*_ is the additional environmental pollution cost (CNY); *U*_*poll*_ is the unit pollution value (CNY/min); *u* is the type of pollutant, *u* = 1,2,3; *c*_*u*,*p*_ is the emission index of *u* (g/kg); *c*_*D*_ is the unit cost of the negative effects of pollution (CNY/g); and *c*^*taxi*^ is the unit taxiing fuel consumption (kg/s).

(3) External cost.

$$C_{total}=\sum_{i=1}^{n}\left(t_{i,queue}\right)\times \left(\frac{1}{n}\sum_{i=1}^{n}\left(p_{i,\;ac}/t_{i,\;ac}\right)+\left(I_{p}/H_{p}\right)\sum_{i=1}^{n}\left(x_{i}\times p_{i}\right)+c_{D}\times c^{taxi}\times \sum_{u=1}^{3}\left(c_{u,\;p}\right)\right)$$ (3)

This approach, which takes into consideration various factors, can analyze data more effectively and is convenient to use.

### Aircraft pushback slot allocation model based on congestion pricing

It is necessary to define the pushback resources before studying congestion pricing. Therefore, the concept of the “aircraft pushback slot” must be introduced. With respect to airport slots [21], aircraft pushback slot allocation can be defined as “the permission given to a carrier to use the full range of airport infrastructure necessary to pushback on a specific time.” Aircraft can be pushed during a specific period after receiving the necessary permissions. The aircraft pushback slot can be described based on the start time, length, and end time. The aircraft pushback slot is actually the time taken by an aircraft to complete the pushback operation. Given the optimization objective of the airport and airlines, in order to effectively utilize pushback resources, the airport allocates pushback slots to aircrafts reasonably, based on a particular mode or credibility, and determines the optimal pushback time for the given aircraft–slot pairs.

#### Assumptions

(1) The conflicts related to taxiing are ignored; (2) the current pushback and taxiing paths are followed without increasing the workload of the controllers; and (3) the choices made by the airlines during each slot are ignored, and the aircraft is pushed at the start time of each slot.

#### Aircraft pushback slot allocation model

The object function of the model is the sum of the surface costs of all the aircraft, *C*. Because the take-off taxiing time is relatively small, it is neglected in the calculation of *C*. The surface time comprises the gate hold time, pushback time, and taxiing out time (including taxiing time and queuing time). Therefore, *C* includes the auxiliary power unit (APU) fuel consumption, *c*_*i*_^APU^; the taxiing out fuel consumption, *c*_*i*_^taxi-out^; the delay cost, *c*_*i*_^*delay*^; and the slot payment, *c*_*i*_^*payment*^. The constraints of the model are primarily the assumptions made as well as the effectiveness, efficiency, and equity of pushback slot allocation. The object function and constraints of the model can be described as follows:

$$\min C=\min\sum_{i=1}^{n}\sum_{j=1}^{m}\left\{\left[c_{i}{}^{apu}\times \left(t_{ij}{}^{gate}+t_{i}{}^{push}\right)+c_{i}{}^{\text{taxi}}\left(t_{i}{}^{taxi}+t_{ij}{}^{wait}\right)+c_{f_{i}}{}^{delay}\;\max\left(0,\left(t_{ij}{}^{gate}-\Delta t_{i}{}^{apply}-10\right)\right)+p_{ij}\right]y_{ij}\right\}$$ (4)

$$such\;that\;\sum_{i=1}^{n}y_{ij}=1,\sum_{j=1}^{m}y_{ij}=1$$ (5)

$$t_{ij}{}^{gate}y_{ij}=\left(f_{ij}^{t_{2}}-f_{i}{}^{apply}\right)y_{ij}=f_{s_{j}}-f_{i}{}^{apply}$$ (6)

$$0\leq \left(t_{ij}{}^{gate}y_{ij}\right)\leq \Delta t_{i}{}^{apply}+40$$ (7)

$$t_{ij}{}^{wait}=\{{}_{2\text{-}\Delta_{io},\Delta_{io}<t_{\min}}^{0,\Delta_{io}\geq t_{\min}}$$ (8)

$$0\leq \left(t_{i}{}^{a-wait}\right)\leq {\left(t^{wait}\right)}^{U}$$ (9)

$$0\leq p_{ij}\leq {\left(p_{ij}\right)}^{\text{U}}$$ (10)

$$\sum_{i=1}^{n}\sum_{j=1}^{m}\left(p_{ij}y_{ij}\right)=C_{total}$$ (11)

where *i* is the aircraft number, *i* = 1, 2, …, *n*; *c*_*i*_^apu^ is the unit fuel consumption of the APU (CNY/min); *c*_*i*_^taxi^ is the unit fuel consumption of taxiing (APU) (CNY/min); $t_{ij}^{gate}$ is the gate hold time (min); $t_{i}^{push}$ is the pushback time (min); $t_{i}^{taxi}$ is the taxiing time (min); $t_{ij}^{wait}$ is the waiting/queuing time (min); $c_{f_{i}}{}^{\text{delay}}$ is the unit delay cost (CNY/min) [22]; $\Delta t_{i}{}^{apply}=f_{i}{}^{t_{1}}-f_{i}{}^{apply}$ (min), is the interval between the scheduled departure time, $f_{i}{}^{t_{1}}$, and the time at which a pushback is applied $f_{i}^{apply}$; *p*_*ij*_ is the price of slot *j* (CNY); *y*_*ij*_ is a decision variable (*y*_*ij*_ = 1 if slot *j* is allocated to aircraft *i*, otherwise *y*_*ij*_ = 0); $f_{ij}{}^{t_{2}}$ is the actual departure time; $f_{s_{j}}$ is the start time of slot *j*, based on assumption (2), $f_{ij}{}^{t_{2}}=f_{s_{j}}$; *t*_min_ is the minimum take-off time interval, which is set by the airport (min); Δ_*io*_ is the interval between the runway arrival times of aircraft *i* and *o* (min); $\Delta_{io}=f_{i}{}^{runway}\text{-}f_{o}{}^{runway}$, where $f_{i}^{runway}=f_{i}^{t_{2}}+t_{i}{}^{push}+t_{i}{}^{taxi}$, $f_{o}{}^{a-runway}=f_{o}^{t_{2}}+t_{i}{}^{push}+t_{i}{}^{taxi}$, and *o* is an aircraft that arrives on the runway slightly before aircraft *i*; (*t*_*ij*_^*wait*^)^*U*^ is the upper limit of the waiting time and depends on the actual departure queue (min); and (*p*_*ij*_)^U^ is the upper limit of the single slot price (CNY).

The objective is to optimize or minimize (4), the surface cost, *C*.

The APU is open while the engine is closed during the gate hold and pushback times; *c*_*i*_^APU^ equals *c*_*i*_^apu^ multiplied by (*t*_*ij*_^*gate*^ + *t*_*i*_^*push*^). The APU is closed while the engine is open during the taxiing out time; *c*_*i*_^taxi-out^ equals *c*_*i*_^taxi^ multiplied by (*t*_*i*_^*taxi*^ + *t*_*ij*_^*wait*^), *c*_*i*_^*delay*^ equals $c_{f_{i}}{}^{\text{delay}}$ multiplied by the delay time, and *c*_*i*_^*payment*^ is the price of the corresponding slot.

Further, expressions (5)–(11) are the constraint conditions.

(5) ensures a single slot is allocated to a single aircraft;

(6) is the formula to calculate *t*_*ij*_^*gate*^;

(7) is the inequality constraint of *t*_*ij*_^*gate*^; the left part ensures non-negativity while the right part is the upper limit and is determined by the delay (*t*_*ij*_^*delay*^ ≤ 30 min):

$$t_{ij}{}^{delay}=f_{i}^{t_{2}}-\left(f_{i}^{t_{1}}+10\right)=\left(f_{i}^{t_{2}}-f_{i}{}^{apply}\right)+\left(f_{i}{}^{apply}-f_{i}^{t_{1}}\right)-10=t_{ij}{}^{gate}-\Delta t_{i}{}^{apply}-10\leq 30$$

(8) is the formula to calculate $t_{ij}^{wait}$;

(9) is the inequality constraint of $t_{ij}^{wait}$; the left part guarantees non-negativity while the right part is the upper limit of the waiting time (*t*_*ij*_^*wait*^)^*U*^.

(10) represents the price constraints for a single slot;

(11) is the equality constraint of *p*_*ij*_; the total price is equal to the external cost.

The model is analyzed in terms of the effectiveness, efficiency, and equity: the effectiveness is reflected in Eqs (5), (7), (8), (9), (10) and (11), which represent the uniqueness of the allocation result, availability of a slot, limit value of the queuing time, and slot price, respectively; the efficiency is defined by the optimization objective function (4); and equity can be interpreted to mean that the model selects the minimization of the total surface cost as the target function and neglects the scale of the airlines, in order to guarantee fair competition.

The above-described slot allocation model corresponds to the problem of determining *p*_*ij*_ and $t_{ij}^{gate}$ for a special target. In this study, we also improved the discrete differential evolution (DDE) algorithm.

#### Improved discrete differential evolution algorithm

In recent years, artificial intelligence algorithms have been used for the optimization of discrete problems [23][24]. The discrete differential evolution algorithm is a stochastic parallel optimization algorithm based on swarm intelligence [25]. It explores the entire population space based on the differences in the information related to the different individuals, performs optimization, and determines the optimal solution using a greedy competition mechanism. The DE algorithm is primarily used with continuous optimization problems. On the other hand, the DDE algorithm has gradually come to play an important role in finding solutions to discrete problems [26]. For the slot allocation problem, the input is fixed and the slots and aircraft are known. Therefore, to determine the slot allocations for a certain period, we propose using an improved discrete differential evolution algorithm (IDDE). The purpose of the IDDE is to improve the differential mutation and crossover operation of the conventional DDE algorithm, in order to enhance the computation speed and convergence performance.

**(1) Differential mutation operation**:

According to the conventional DE algorithm, the mutation method is given as follows [27]:

$$v_{i,g}=x_{r_{0},g}⊕F⊗\left(x_{r_{1},g}-x_{r_{2},g}\right)$$ (12)

where *g* is the number of evolutional generations, *r*_0_ ≠ *r*_1_ ≠ *r*_2_ ≠ *i*, *i* = 1, 2, …, *N*; and *F* is a scale factor (*F* = 0.5). (12) contain two parts: the temporary vector, *δ*_*i*,*g*_, and the mutant individual, *v*_*i*,*g*_.

$$\begin{array}{l} \delta_{i,g}=F⊗\left(x_{r_{1},g}-x_{r_{2},g}\right)\Leftrightarrow \\ \delta_{i,j,g}=\left(x_{r_{1},j,g}-x_{r_{2},j,g}\right)\mathrm{sgn}\left(\max\left(0,F-rand\left(0,1\right)\right)\right)=\{{}_{0,otherwise}^{x_{r_{1},j,g}-x_{r_{2},j,g},rand<F} \end{array}$$ (13)

where sgn(⋅) is the sign function, which returns to an integer set (1, 0, -1).

$$\begin{array}{l} v_{i,g}=x_{r_{0},g}⊕\delta_{i,g}\Leftrightarrow \\ v_{i,j,g}=x_{r_{0},j,g}⊕\delta_{i,j,g}=\mathrm{mod}\left(\left(x_{r_{0},j,g}+\delta_{i,j,g}+N\right),N\right) \end{array}$$ (14)

where mod(⋅) is the modulus operator.

The vectors of *v*_*i*,*g*_, which was calculated by (14), may appear “0” (e.g., 0, 2, 3, …, *N*), but the solution vectors are composed of the integer set (1, 2, …, *N*); 0 is out of the search area of the solution vectors, in order to limit the calculation results to the search area. In this study, we made the following modification [28]:

$$\begin{array}{l} v_{i,g}=x_{r_{0},g}⊕\delta_{i,g}\Leftrightarrow \\ v_{i,j,g}=x_{r_{0},j,g}⊕\delta_{i,j,g}=\mathrm{mod}\left(\left(x_{r_{0},j,g}+\delta_{i,j,g}+N-1\right),N\right)+1 \end{array}$$ (15)

After the improvements have been made, the solution vectors are limited to the search area, and the individual target vectors can be obtained. However, this also generates repeated integers, resulting in infeasible solutions. Therefore, it is necessary to delete these, in order to form feasible aircraft–slot pairs.

**(2) Crossover operation**:

The crossover operation generates a trail individual, *u*_*i*,*j*,*g*_, by combining a mutant individual, *v*_*i*,*g*_, and a target individual, *x*_*i*,*g*_. This is done using the following procedure:

Step 1: generate random numbers, *rand*(0,1). Further, *C*_*r*_ is the crossover rate; if *r* < *C*_*r*_, then *u*_*i*,*j*,*g*_ = *v*_*i*,*j*,*g*_, *j* = 1,2,⋯,*N*;

Step 2: if the dimension of the *j*-th component of *x*_*i*,*g*_ is the same as that of *u*_*i*,*g*_, retain the component *u*_*i*,*g*_;

Step 3: delete the repeated integer of *u*_*i*,*g*_ (random permutation; the integer in this permutation represents the serial number of the repeated integer);

Step 4: put the rest of the integers of *u*_*i*,*g*_ in the vacant positions (to avoid bias in the selection of the vacant positions, generate a random permutation; the integers in this permutation represent the serial numbers of the integers in *x*_*i*,*g*_, which is different from *u*_*i*,*g*_).

The test vector is determined based on both the target vector and the mutant vector after crossover and is used to generate a 1–1 aircraft–slot pair.

Scientific analysis of IDDE: correct the solution vector that spills over the normal range, restrict it to the search space by translating the formula for a mutant individual during the differential mutation operation, and present the corresponding crossover strategy to remove the repeated integer in the solution vectors. These improvements can prevent invalid operations and increase the operation speed.

## Simulation and Analysis

### Data preparation

#### Basic data

A simulation was performed for Xinzheng International Airport, China (13:00 to 14:00, April 15, 2016).

The simulation conditions were the following:

Runway configuration: 12L|12R; scheduled pushback of 17 aircraft (Table 1); $f_{i}{}^{t_{1}}$, *c*_*i*_^*taxi*^, and $f_{i}^{apply}$ are known; and *t*_*i*_^*push*^/*t*_*i*_^*taxi*^ is the ratio of the pushback/taxiing path length and the pushback/taxiing speed. All the aircraft were midsized, *c*_*i*_^apu^ = 5 CNY/min, and $c_{f_{i}}{}^{\text{delay}}$ = 5 CNY/min; In order to ensure the safety of take-off, the airport set *t*_min_ = 2 min and determined unit length of slot = 3.5 min, (*t*_*ij*_^*wait*^)^*U*^ = 10 min, and (*p*_*ij*_)^U^ = 500 CNY. The initial aircraft data is listed in Table S1 Dataset, coming from Henan Province Airport Group.

**Table 1:** Aircraft data.

No.	Flight Number	$f_{i}{}^{t_{1}}$	*t*_*i*_^*push*^ (min)	*c*_*i*_^*taxi*^ (CNY/min)	*t*_*i*_^*taxi*^ (min)	$f_{i}^{apply}$
1	ZH9637	13:00:00	2.14	29.76	13.86	12:57:00
2	CZ6369	13:05:00	1.11	29.37	10.37	13:02:00
3	8L9954	13:10:00	1.76	29.37	14.77	13:06:00
4	PN6281	13:10:00	1.23	29.76	16.82	13:07:00
5	GS6508	13:15:00	1.11	29.76	15.54	13:11:00
6	CZ6359	13:15:00	1.11	29.76	10.29	13:12:00
7	ZH9435	13:20:00	1.89	29.37	14.54	13:17:00
8	ZH9437	13:25:00	1.89	29.76	14.25	13:21:00
9	FM9390	13:25:00	1.37	29.37	7.77	13:22:00
10	JD5400	13:30:00	1.89	29.76	13.96	13:26:00
11	KY8258	13:30:00	1.82	29.37	13.57	13:27:00
12	CZ6439	13:40:00	1.58	29.37	10.29	13:37:00
13	CZ3592	13:45:00	3.14	29.37	10.29	13:41:00
14	FM9296	13:45:00	1.37	29.37	11.51	13:42:00
15	GS7523	13:50:00	1.37	29.76	11.19	13:47:00
16	CZ3495	13:55:00	1.11	29.37	10.20	13:51:00
17	ZH9149	13:55:00	2.61	29.37	13.67	13:52:00

#### Calculation of external cost of congestion

All the departure aircraft were midsized and of the same type. The various parameters used are listed in Table 2.

**Table 2:** Parameters used to calculate external cost.

Parameter	Value	Parameter	Value	Parameter	Value
*t*_*i*,*queue*_	3.41 min^a^	*H*_*p*_	48192 min^d^	*c*^*taxi*^	0.19 kg/s
*p*_*i*,*ac*_	821.2 CNY^b^	*x*_*i*_	17 aircraft	*c*_1,*p*_	1.095 g/kg
*t*_*i*,*ac*_	221.97 min^c^	*p*_*i*_	135 passengers^e^	*c*_2,*p*_	25.588 g/kg
*I*_*p*_	25576 CNY^d^	*c*_*D*_	10 CNY /kg	*c*_3,*p*_	4.601 g/kg

^a^Taxiing out time at peak hours minus that at off-peak hours.

^b^Statistical data for period from March to June 2016 (source: China Business News).

^c^Boarding time was 30 min earlier than the scheduled departure time, and the approach time at arrival airport was 20 min.

^d^*I*_*p*_ is the average annual income of all urban residents. (Source: Statistical bulletin of national economic and social development in Henan province).

^e^Passenger capacity: 150 passengers, peak load factor: 90%.

Then, using Eq (3), the equality constraint of the slot price, *C*_*total*_, could be determined (2082 CNY).

### Simulation

**Fig 2:**
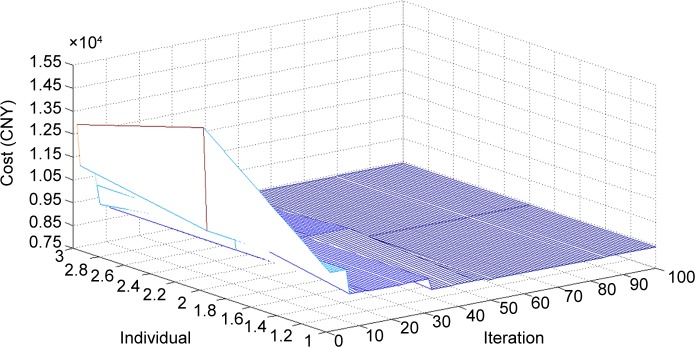
Optimization process of IDDE algorithm as implemented in MATLAB.

The model was solved using the DDE and IDDE algorithms separately. The run times were 56.017 s and 30.967 s, respectively. The optimization process for the IDDE algorithm is shown in Fig 2.

As can be seen from Fig 2, the surface cost minimizes after 35 iterations. Thus, the IDDE algorithm shows a high efficiency, resulting in the lowest costs and operation time. The results of the simulation performed using the IDDE algorithm are shown in Table 3.

**Table 3:** Results of simulation performed using IDDE algorithm.

Aircraft	Slot	*p*_*ij*_ (CNY)	*t*_*ij*_^*gate*^ (min)	*t*_*ij*_^*wait*^ (min)	*C*_*i*_ (CNY)
1	1	158	3.00	1.23	633
2	2	158	1.50	0.00	476
3	3	87	1.00	0.00	535
4	4	106	3.50	0.00	630
5	6	162	6.50	0.49	677
6	5	81	2.00	0.00	403
7	7	34	4.00	0.00	490
8	9	85	7.00	0.00	554
9	8	184	2.50	0.00	432
10	10	221	5.50	0.00	673
11	11	19	8.00	0.00	467
12	12	210	1.50	1.77	580
13	13	188	1.00	0.00	511
14	14	173	3.50	0.00	535
15	15	66	2.00	0.00	416
16	16	132	1.50	0.00	445
17	17	18	4.00	0.00	453

As can be seen from the *p*_*ij*_ values, only 2 slots have a price greater than 200 CNY, with 47% of the slots being cheaper than 100 CNY; these low prices would be readily accepted by the airlines, making the method easy to implement.

### Analysis

The following strategies were compared: grandfather rights (U), pushback slot control (V), and pushback slot control based on congestion pricing (W).

#### Surface cost

**Fig 3:**
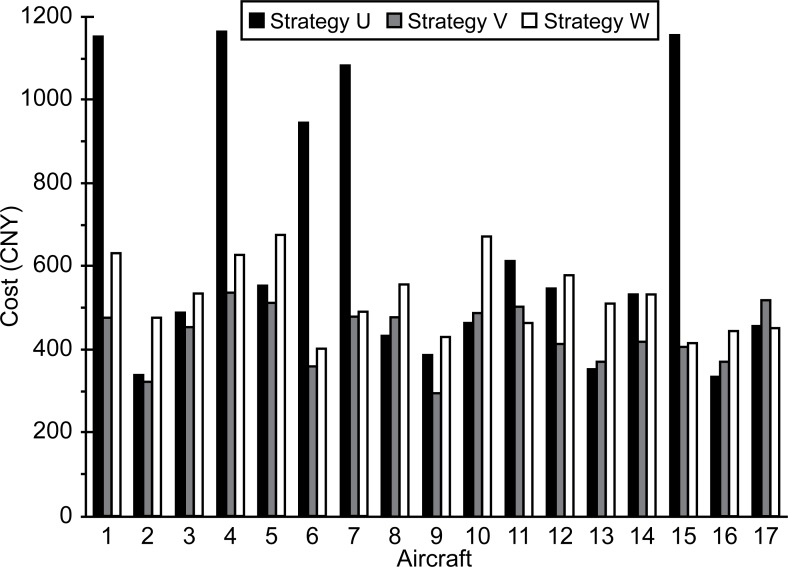
Surface cost for each aircraft.

**Fig 4:**
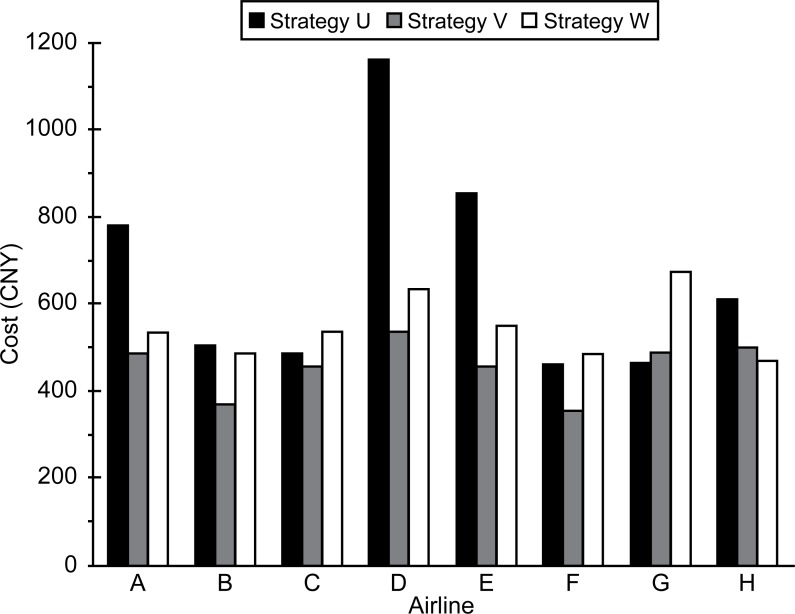
Average surface cost for each airline.

The surface costs for each aircraft and airline under the three strategies are shown in Figs 3 and 4.

Figs 3 and 4 show that the highest total cost for each aircraft and airline were obtained when strategy U was used. This was followed by strategy W. The reasons for this are as follows: (1) U has the highest delay cost and (2) under W, usage fees are charged to the airlines. The increased costs can be thought of as the monetary compensation paid by the airlines for the negative effects (congestion and pollution) caused when they push aircraft during rush hour.

Taking the average cost of each airline as an example, it can be seen that W is only 88 CNY more expensive than V and is far cheaper than U.

#### Delay

For a comparative analysis, we analyzed the pushback delay (hereinafter referred to as the delay) instead of the simple delay.

**Fig 5:**
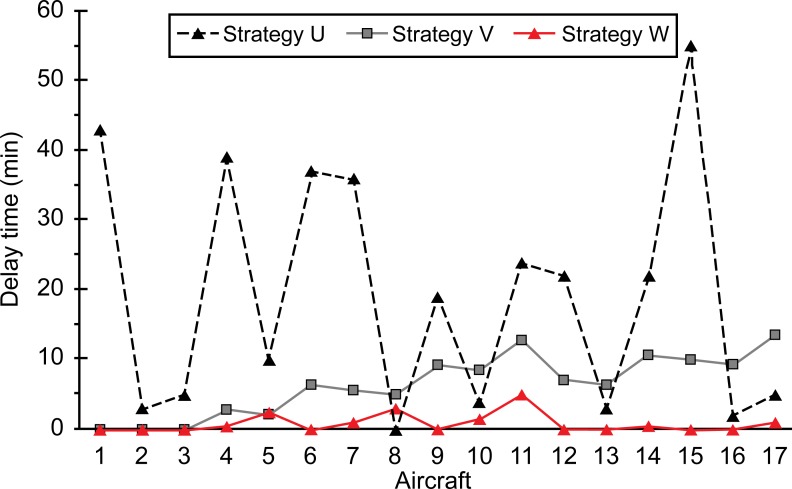
Delay time for each aircraft.

**(1) Delay time:** The delay time for each aircraft is shown in Fig 5.

As shown in the Fig 5, the delay in the case of strategy U was significant (maximum of 55 min, average of 19.4 min). On the other hand, the average delay in the case of V was 6.5 min and 67% lower than that for U. Further, the average delay for W was 1 min and 86% lower than that for V. Therefore, strategy W is the best suited for optimizing the delay time. The standard deviation in the delay time for U was 16.78, indicating that the degree of discretization was very high. The standard deviations for V and W were 4.21 and 4.21, respectively. Thus, slot allocation using strategy W would be the most equitable.

**Fig 6:**
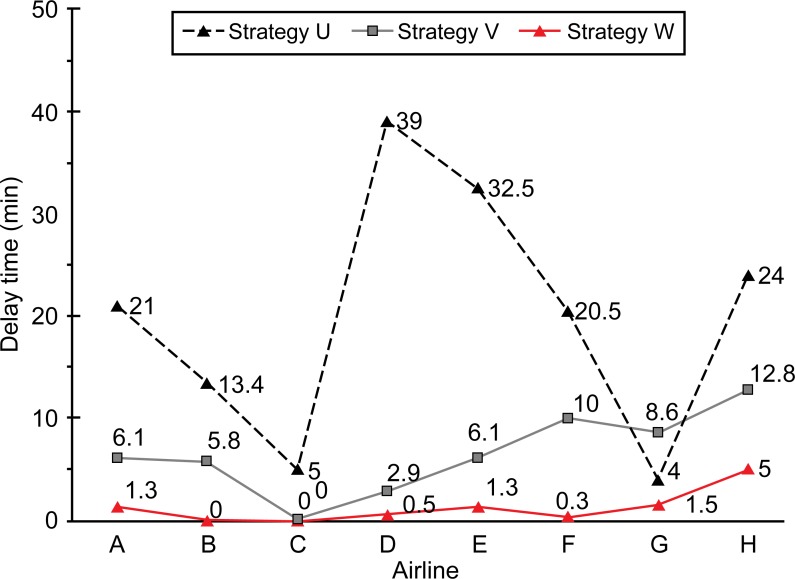
Average delay time for each airline.

The average delay time for each airline is shown in Fig 6.

As shown in Fig 6, the delay in the case of strategy U was 5–39 min, with the average delay being 19.9 min. The delay in the case of V was 0–12.8 min, with the average delay being 6.5 min; this was 67% lower than that for U. Finally, the average delay in the case of W was 1.2 min, which is 81% lower than that for V. Therefore, strategy W is the best suited one for optimizing the delay time of the airlines.

**(2) Delay distribution:** Next, we calculated the proportion for which the aircraft/airlines experienced the different delays. If the proportion of small delays is low and that of large delays is high, the effect of the delay distribution can be considered weak. On the other hand, if the proportion of small delays is high and that of small delays is low, then the effect is optimal.

**Fig 7:**
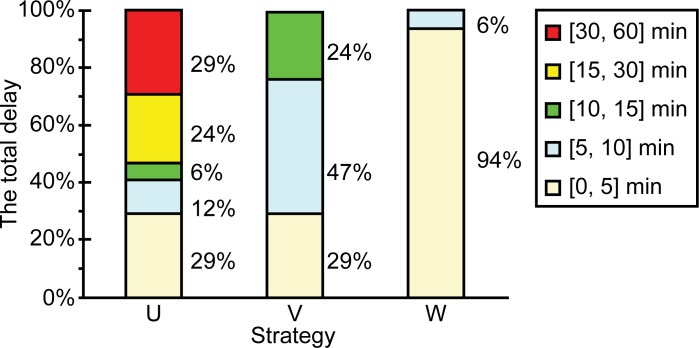
Stacked column chart showing the proportions for which the various delays were experienced by the aircraft.

A stacked column chart of the proportions for which the various delays were experienced by the aircraft is shown in Fig 7.

The delays could be divided into small delays (0–10 min), moderate delays (10–15 min), and large delays (15–60 min).

For each aircraft, the delay distributions for U, V, and W were 41/6/53, 76/24/0, and 100/0/0 respectively (Fig 7). Hence, the effect of strategy U was the worst while that of strategy W was the best. Thus, it can be concluded that W results in the highest degree of optimization of the delay distribution of the airlines.

**Fig 8:**
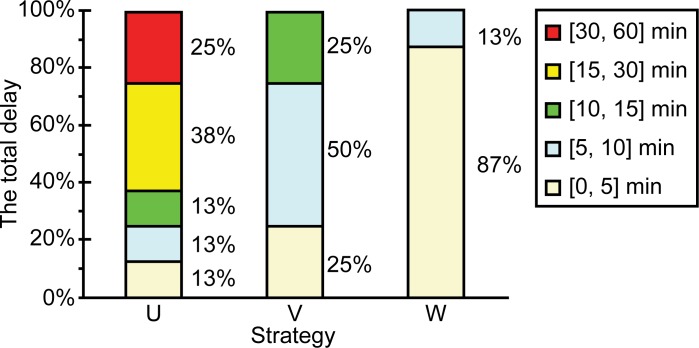
Stacked column chart showing the proportions for which the various delays were experienced by the airlines.

Fig 8 shows the stacked column chart for the proportions for which the various delays were experienced by the airlines.

For each airline, the delay distributions for strategies U, V, and W were 26/13/63, 75/25/0, 87/13/0 (Fig 8). Thus, in this case too, the optimization ability of strategy W with respect to the delay distribution was the highest.

## Conclusions

In this study, the problem of optimizing the aircraft pushback time allocation process was studied, in order to determine the optimal strategy for reducing surface costs and delays and hence relieving congestion.

The concept of the “external cost of surface congestion” was proposed, and an expression for quantizing it was developed and used as the basis for congestion pricing. Then, with the aim of ensuring the lowest cost, an aircraft pushback slot allocation model based on congestion pricing was proposed, and an improved DDE algorithm for the aircraft pushback slot allocation model was designed. Finally, a simulation analysis was performed to confirm the feasibility and effectiveness of the proposed model and algorithm.

The primary conclusions of the study can be summarized as follows:

(1) The computation time of the IDDE is 44.7% shorter than that of the DDE. Further, the IDDE has a faster convergence rate and smoother convergence process.

(2) The surface cost could be decreased from 666 CNY to 456 CNY after slot control, indicating that slot control can significantly reduce costs. Although the cost of slot control based on congestion pricing increased to 544 CNY, it was still much lower than that of grandfather rights. The increased amount would be readily acceptable by airlines, ensuring the acceptability of the proposed method.

(3) Slot control reduced the delay time for single aircraft, with the average delay decreasing by 67% (from 19.4 min to 6.5 min). Further, slot control based on congestion pricing reduced it even more, by 86% (from 6.5 to 1.0 min). The standard deviation also decreased, from 16.78 to 1.37, indicating that slot control based on congestion pricing can significantly reduce delays, leading to equitable allocations.

Further, slot control based on congestion pricing resulted in relatively low delay times for all aircraft and airlines (10 min). Thus, it can be employed for optimizing the delay distribution.

(4) The analysis results showed that, when several aircraft belonging to an airline compete for slot resources, slot control based on congestion pricing is a favorable option. In practice, most airlines have to push more than one aircraft during peak hours. Thus, the proposed method would be feasible under these conditions.

The above-described analysis showed that, compared to grandfather rights, the proposed pushback slot control strategy based on congestion pricing can significantly reduce the queuing time, ease congestion, reduce delays, and optimize the delay time distribution. Further, it does not result in increases in the surface cost. From the perspective of resource allocation, the paid use of pushback slot resources during rush hour is highly recommended. This part of the income can be used for infrastructure construction, which would aid in the sustainable development of the airport surface resources.

The proposed model can be used to formulate a pricing strategy for pushback slots when considering decision-making by the airport and airlines. Under this strategy, the pushback slot usage fees are charged to the airlines, the toll rate is low, and the airlines can select slots based on a surface cost analysis.

Thus, the results of this preliminary study confirmed the feasibility of using a price mechanism in aircraft pushback management as a novel way of solving the pushback slot allocation problem.

## Supporting Information

S1 Dataset Aircraft and slot data. (XLSX)
